# Commentary: expectations for global health program prioritization from a selection of international students studying at a European university

**DOI:** 10.1186/s12992-016-0193-5

**Published:** 2016-09-22

**Authors:** John Quinn, Vít Lidinský, Venu Rajaratnam, Marta Kruszcynski, Tomas Zeleny, Vladimir Bencko

**Affiliations:** 1 Prague Center for Global Health, Institute of Hygiene and Epidemiology, First Faculty of Medicine, Charles University, Studnickova 7, Prague, 121 00 Czech Republic; 2 Pražská znalecká kancelář, s.r.o. IC: 48910660, Prague, Czech Republic; 3 Institute of Hygiene and Epidemiology, First Faculty of Medicine, Charles University, Prague, Czech Republic; 4 Institute of Economic Studies, Faculty of Social Sciences, Charles University, Prague, Czech Republic

**Keywords:** Global health curricula, Global health diplomacy, Medical education, Medical students and staffing, Medical meetings

## Abstract

**Background:**

Some university curricula struggle to present evidence-based promotion of global health principles and global health diplomacy within an undergraduate setting. The *de facto* global health paradigm has experienced significant stress and pressure from epidemics, war and violence, climate change and resource challenges. These stressors may lead to increased morbidity and mortality, in turn requiring medical professionals to play a larger role in global health action across borders.

**Methods:**

In the academic year 2014-2015, an English-speaking international medical school promoted a global health forum with pre-course readings and a pre-attendance quiz. All students from the university were invited to attend and the event was not mandatory.

**Results:**

The one-day-event culminated in expert speakers, discussions and a post-event questionnaire to gauge students’ reactions and expectations as future physicians regarding the most pressing global health topics. Emphasis was also placed on what future doctors foresee as pressing issues in forthcoming global health policy and programming.

**Summary:**

This paper is a brief commentary of the Global Health Forum in Prague 2014, and presents novel results from a post-event student questionnaire, with conclusions provided by students on innovative global health policy.

## Background

Headlines concerning beheadings by the Islamic State (ISIS) in the Middle East, rockets aimed at and incursions into the Holy Land, borderless morbidity and mortality caused by the Ebola virus and hybrid war in Ukraine illustrate the challenges that global health institutions face today. The scale of such crises may leave major response efforts scrambling in a bid to respond to violence and disease. Reduced resilience to these shocks and increasing crises challenges global health actors and stakeholders in their mandate to provide best practices for all [1, 2]. This challenge is exacerbated by the absence of no clear global health governance structures that can help cushion these shocks. Global institutional capacity may be stalled and best practices promoting evidenced-based policy may be lacking, leaving the venue of global health projects and programming at risk of fragility and failure [3].

Disease does not discriminate and knows no borders. The realities of the Ebola virus and more recently, the Zika virus, as well as potentially unknown threats, forces us to ask whether we are prepared for dealing with such threats. Although resources are scarce and funding minimal, it should be our collective responsibility and shared resolve internationally to adjust and form an appropriate global health infrastructure with the aim of recognizing, responding and managing crises, without ignoring current endemic problems.

Despite the uneasy abatement of the Ebola epidemic and short gains in other communicable diseases such as tuberculosis (TB) and malaria, and until recently, polio, many issues remain. The international community and global health policy makers have failed to provide an organized, systematic and sustainable response to recent catastrophic infectious disease outbreaks, complex emergencies of war and social unrest and worsening environmental degradation. If the global health community does not achieve a rapid and steep learning curve, these crisis events, as well as mortality rates, may increase and repeat logarithmically [4].

However, it must be noted that when the global health community and its seemingly disparate pool of actors are able to coalesce and focus on evidence- based strategies and policy, success has been achieved in the form of reducing morbidity and mortality. These advancements are not only the result of sound evidence-based policy, but also due to the presence of standardized global health curricula and education and training for health practitioners [5]. The purpose of the *Global Health Forum in Prague* is to support standardized curricula in global health and to instill awareness for future global health leaders.

## Selected global health problems list

### Ukraine

A humanitarian disaster is arising in Europe’s backyard. According to WHO data, mid-summer 2016 saw more than two million displaced people, an estimated 9250 or more killed, over 27 000 wounded, a fully-packed civilian airliner detonated at 36 000 feet and the sovereign nation of Ukraine occupied by foreign tanks and foreign troops [6]. An immediate public health crisis concerning emergency medicines subsists, while access to vaccines such as those for polio and tetanus is absent; basic services such as water, hygiene, shelter and primary or emergency medical care is not available for those caught in the crossfire of an uncertain future, where tanks with unmarked troops keep advancing on poorly demarcated occupied zones.

### Violent extremism: the Middle East

ISIS has infiltrated multiple countries and has murdered thousands through wanton acts of violence and torture defined as genocide by the UN. With this, women’s health and empowerment have been decapitated and communities are barred access to primary and emergency healthcare services across borders.

ISIS is armed and possesses the financial and logistic support to continue killing, thus deteriorating the region into further health and security chaos. Escalation of violence in Syria and an increased Russian military presence and operations may be pushing almost 1 in 2 Syrians to flee to European soil. This is in addition to other fragile states in the Middle East and across the Sahel in Africa that are sending refugees and displaced peoples northward due to social and political unrest and instability. These stressors collectively exacerbate children’s and women’s health, intensify gender-based violence and exacerbate communicable and non-communicable disease (NCDs) [7]. ISIS highlights the requirement for global health governance and mandate, especially in ungoverned spaces and its relationship with state and non-state actors.

### Ebola

Meanwhile, in West Africa, the well-documented Ebola virus has reared its head and has swiftly crossed borders, cultures and societies. The mechanisms that enabled such wild proliferation are currently under study, yet preliminary reports by the WHO and third parties reveal a slow and half-hearted global response to the crisis, possibly related to governance, persistent funding inadequacies, a dearth of well-trained medical staff and the conclusion that such an outbreak can happen again with very few safeguards in place and no lessons learned from previous experience [4, 8]. It should be noted that in the month of September 2015, newly confirmed cases of the Ebola virus were still being reported in both Guinea and Sierra Leone, with associated fatalities [9]. And in April 2016 another case was confirmed in Liberia. The Ebola outbreak is not over and the spread of the Zika virus has not yet reached its full scale.

All of the above crises and emergency threats exist above and beyond the pre-existing crises of multidrug resistant tuberculosis (TB), malaria, diarrheal illness and the mortality of under-five-year-olds, HIV/AIDS, many neglected tropical diseases and the current proliferation of non-communicable diseases (NCDs) sweeping an overall unhealthy, smoking, sedentary global population with a poor diet in both least-developed and most developed countries alike [10].

However, it should be noted that global health infrastructure has had some success in collectively combating some of these global health concerns through solid leadership and transparent institutions. Indeed, some successes have managed to reduce certain diseases and social behaviors. For example, the establishment of the Millennium Development Goals (MDGs) set forth an agenda to prioritize and devote funding to very specific categories of global health threats.

The above endpoints and metrics have had varying degrees of success and as such, the new and revised Sustainable Development Goals (SDGs) will set a framework and path for sustainable development in multiple health and development streams for future generations, in effect bringing global health governance into a framework where actors and stakeholders can all be up to date about the same policy. The governance and focus of these metrics for the SDGs are still being drafted mid-2016. Despite these significant successes, however, many issues remain, and some old threats such as polio are once again finding their way back into at risk communities and populations.

## The institution

Charles University, First Faculty of Medicine offers both undergraduate and graduate degree programs in English and in Czech. The Prague Center for Global Health is a research center based in the Institute of Hygiene and Epidemiology, which focuses on human and health security in conflict and post-conflict settings, and researches many of the above-mentioned global health concerns in Central Europe.

## Methods

In an effort to focus on pressing global health topics, the Prague Center for Global Health held its second biennial Global Health Forum in Prague in 2014. The primary theme of the Global Health Forum 2014 was prevention in conflict areas, pandemics and the promotion of health security. Students were given pre-course readings and a pre-event quiz. Ethical approval was granted for the study by the Departmental Internal Review Board (IRB) of the Institute of Hygiene and Epidemiology, First Faculty of Medicine, Charles University. No reference numbers were given.

During the event, all participants submitted verbal consent for the results of the Forum and online quiz to be published, and also completed written informed consent as part of the survey when reporting their individual responses. On the day of the event, two hours were spent with speakers and professors reviewing key questions and topics from the readings prior to keynote presentations. Following the presentations, a videoconference with Dr. Greg Martin and full panel discussion with all speakers reviewed student questions and perspectives. Upon conclusion of the Forum, a post event questionnaire was sent via email to all participants and speakers (see “Questionnaire” details below.

### Speakers

The speakers and topics of the invited presenters included:

- Melaura Myers, MD, medical resident – recorded video presentation: *Rural pediatric health in Tanzania*. This talk discussed the need for primary prevention in rural communities and specifically, the burden of disease in rural Tanzania and access to vaccines.
- Dr. Greg Martin, Editor in Chief at *Globalization and Health* – recorded video presentation: *Fragile and failed nation states and health*. This robust and highly effective recorded presentation put into practical terms the health challenges and barriers faced by those who reside in fragile and failed nations concerning infectious and noncommunicable diseases (NCD).
- Dr. Alena Šteflová, Head of the WHO Country Office (Czech Republic) at the World Health Organization: *Ebola and WHO*. This presentation discussed the extremely serious Ebola outbreak of 2014, the WHOs’ response and budgetary commitments to the outbreak, as well as present and future challenges concerning global health security threats.
- Diplomat Elaine Kelley, US Department of State: *The Ebola outbreak and US response*. This presentation focused not only on the United States’ commitment to global health, but also described how current programs like the President’s Emergency Plan for AIDS Relief (PEPFAR) can be dovetailed into further global health programming for ensuring global health security.
- Prof. David McIntosh, Honoree senior lecturer at Imperial College London: *Abbreviated presentation on vaccine in conflict and disaster and rapid vaccine development*. This cutting-edge discussion on new vaccines, technologies and rapid access to tailored vaccines was cut short due to time constraints; however, the basics were highlighted.

### Questionnaire

The questionnaire was offered through a paid Survey Monkey (surveymonkey.com) account supported by Mercy University Hospital (MUH) in Cork, Ireland, under Dr. Iohmar O’Sullivan, and is available at: http://www.surveymonkey.com/r/PRG_CUP_GHF_2014. All results, as well as individual responses, were compiled and reviewed for qualitative and quantitative measures using the surveymonkey.com software. The mix of questions was extremely broad and reflected the nature of global health, as well as the speakers’ topics and the pre-course reading.

### Student attendance and access

The Global Health Forum 2014 in Prague was interdisciplinary, apolitical, free of charge, free of private sponsorship and the organizers and supporters promoted no political or governmental agenda of any kind. All students in both the Czech and English language groups were invited to attend via posts on Facebook, LinkedIn, the official University Website and through word-of-mouth. Attendance was not mandatory; all student years across Charles University were invited to attend. In terms of ethical considerations, the students surveyed were not asked for their country of origin, country of residence or ethnic background. The main qualifiers were students’ year of medical study and their reactions to the speakers and their topics.

Due to the social and political nature of the events to be discussed, diplomatic missions to the Czech Republic from Israel, Palestine, Ukraine and Russia were invited. Russia declined to participate in an official capacity and no other correspondence was received from any other country or their representatives.

## Results

A total of 47 respondents completed the online questionnaire. The Global Health Forum in Prague 2014 was attended by 56 international undergraduate and graduate students from Charles University, Faculties of Medicine and across different years of study. General medical students made up the majority of attendees at 89 %; of these, 67 % were in the final two years of their medical degree with only 4 % first-year medical students in attendance. Postgraduate students represented 3 % of attendees. Of all the students in attendance, 7 % were dental students. No ethnic, country of origin or residence data were collected; anecdotally, the university’s diverse student body comprises more than 60 nationalities and countries who will subsequently practice medicine and dentistry globally [11].

Among respondents, 37 % reported having an interest in global health, health policy or conflict medicine prior to the Forum. The majority (48 %) reported that they were “somewhat” interested, while 14 % of respondents were neutral, with no particular interest for or against global health. After attending the Global Health Forum, respondents reported an overall positive experience, with more than half of attendees expressing an increased interest in global health and health policy; 18 % of respondents expressed a new interest in these areas and 33 % expressed having a renewed interest in the field after attending and participation at the event.

### Future medical practice

Overall, the Forum provided clarity to 33 % of students who felt that the Global Health Forum “would influence or change their future decision [regarding] specialty training or practice”. According to the responses provided by dental students in attendance, they were least likely to be influenced by the Forum with regard to their future career goals. The added interest affected by the health forum was relevant to general medical student academic progression. Some students concluded in open-ended questions that the Forum should continue to be a resource for general medical students, and offer improvements in terms of addressing the relevance of global health issues to future medical and dental students.

### Prevention medicine

When asked specifically, “[about] remote medicine in rural areas in Tanzania, do you find a connection between vaccine preventable illnesses like pneumococcal pneumonia and increased morbidity or mortality in rural and remote areas globally?”, 68.18 % of respondents answered ‘yes’, 22.73 % responded, ‘I don’t know’ and 9.09 % answered ‘no.’

The open-ended question, “What strategy would you use to challenge cultural perceptions to vaccines in an era where anti-vaccine maxims are growing in popularity and herd immunity may be decreasing with negative effects?,” elicited overwhelming responses with an emphasis on patient empowerment and education related to the benefits of vaccines and primary prevention. Some detailed and specific responses are listed below:

*“More governmental involvement in school, educating students and kids about vaccines. Also reaching out to small ethnicities that might not speak the language of the country they live in.”*

*“One strategy that could be implemented is to bring to the first world countries attention of how preventable diseases and infections due to vaccines affect developing nations, and specific populations, that do not have access to such vaccine. This could provide a real life contrast to what could happen in first world countries if this “anti vaccine” trend continues. I think people related more to situations and topics if they can put a name and a face to certain issues. Also implementing some epidemiological study/research research study to find out why anti vaccinators have this preconception about vaccines and how the trend is growing in popularity in order to target these specific issues.”*

*“I will educate my patients on the importance of vaccination, and give them examples of how dangerous it can be when parents don’t vaccinate their kids. I will explain to them how herd immunity works, and what their responsibility is.”*

*“Three vaccine delivery strategies: 1) school-based vaccination; 2) health-centre-based vaccination; and vaccination combined with other health interventions.” [sic]*

*“Showing the data about the reemergence of preventable illness because of absence of vaccination and emphasizing the risk and complication of every disease and further increasing the education again regarding how to prevent the diseases.”*

### Ebola

When asked, “In the setting of an epidemic of communicable disease such as Ebola, there is increasing pressure to produce ‘vaccines on demand’. [N]ame three defining factors when it comes to stemming an epidemic of this nature”, many student responses focused on quarantine and travel restrictions from endemic zones. Specific responses were longer and more detailed, and offered student-based insights into this pressing global health concern:

*“There are many defining factors when it comes to stemming an epidemic… I believe that socioeconomic issues are on the forefront of the EBOV (*Ebola Virus*) epidemic; such as was war, poverty, and poor health infrastructure:*

- *1. Poor health Infrastructure has definitely helped in the spreading of EBOV. If there was a more sophisticated health care system, I believe that speed of EBOV spread might have been diminished.*
- *2. War - the region of West Africa has been in civil unrest, this causes mass movement of people, refugee, and vulnerable people, allowing for both contracting EBOV and spread.*
- *3. Culture Issues - ranging from burial practices -(where certain groups of people do not believe in cremation - which is necessary procedure in the anti-epidemic protocol) to traditional medical practices and also fear of modern medicine and health care practice.”*

*“Mode of transmission, animal reservoir, political factors-international cooperation to prevent and treat disease.”*

*“Economic and public health/medical perspectives play an important role in the policy process.”*

*“Cooperation of major producers to receive the very expensive production costs. Mobilize and organize resources, doctors and medication, development of vaccines and health equipment. Treat the infected in hospitals and try to contain the infection and setup regimes in order to prevent increased spreading.” [sic]*

### Financial response

When students were asked, “Do you think the funding efforts by the international community are sufficient [for] controlling the epidemic?”, 4.76 % answered ‘more than sufficient,’ 47.63 % answered ‘sufficient but can do with additional funding’, 38.10 % said ‘barely meeting the demand’ and 9.52 % answered ‘I have no idea.’

### State fragility and health security

When asked about state fragility, i.e., “[D]oes state fragility or failure better enable the spread of pandemics and infectious disease?”, 100 % of students replied, ‘yes.’ When asked, “Do you think sending military assets to a pandemic zone will benefit the relief effort and lead to eradication of the disease?” 60 % replied ‘yes,’ 10 % ‘no’ and 20 % replied ‘these issues are not linked.’

Among the respondents, 10 % chose ‘other’, with one student stating: *“I do not think it’s a question of yes or no… [It depends] on the type of epidemic and the population. On one hand, the “military assets” has the advantage of stopping the spread and local control but on the other hand there are ethical problems with it. I think that good education should combine always with military entrance.”*

When asked, “Despite many pundits claiming US power is declining globally, no other single country has proposed a comprehensive response plan to the epidemic (Ebola). Is the response by US forces and public health care adequate to stop the spread, 10 % of students said ‘yes,’ 45 % responded ‘yes, but more can be done,’ 30 % said ‘no’ and 15 answered ‘no comment.’

### Climate change

Some authors purport that environmental buffer systems have been pushed to their limit as a result of climate change and degradation, and that there may be a connection between these events and infectious disease [12]. When students were asked, “Based on this hypothesis, do you think there may be more or less infectious disease outbreaks as environmental degradation continues to be challenged?”, 47.62 % responded, ‘yes,’ 19.05 % said ‘no’ and 33.33 % were ‘unsure.’

### Hybrid war in Ukraine

More than 9250 people have been killed in Ukraine since hostilities began in 2014 with the Crimean Peninsula being annexed by Russian forces. More than two million people have been displaced from their homes and cannot access basic healthcare, water or basic hygiene services. Based on this information, students were asked, “How can NATO and more economically developed countries address the Ukrainian humanitarian disaster to encourage global health?” Responses were open-ended and focused on sending more aid, placing more political and economic pressure on Russia in a bid to stop its support of violent attacks, and even, *“by eating Putin for breakfast.”* Further specific responses by student participants that focused on global health policy by student participants included:

*“Countries have put sanctions on Russia, which to an extent is a way of showing their disapproval with Russia's action. But sanctions also affect the citizens of Russia, who have not chosen to go to war in Ukraine. And if anything, may lead to financial instability in the homes of Russian families, and from their trickling down to the clear connection of financial and social instabilities linking to worse health. Although I do believe sanctions are necessary. I do think economically developed countries need to place greater emphasis on becoming more proactive on the ground, providing proper health facilities in war zones, having safe zones accessible to all the victims of war, having water zones be safe zones and also helping allocate displaced peoples and actively communicating with all political parties in trying to resolve this issue.” [sic]*

*“The main problem as I understood that the “Humanitarian aid” could not enter or reach to the places and people in Ukraine … The effort in this days to encourage global health should be focused on clean water, food and medical supplies to “prevent” a post- disaster effect of low hygiene situations etc…” [sic]*

*“Boycotting Russia in order to inflict a bit of pain (financially) is a further possibility and sending a team to help rebuild the foundational in health; and it can be done not by sending professionals (which obviously not everyone wants to be benevolent) but maybe by sending students as part of the education of their course … construction engineering etc…” [sic]*

## Discussion

Global health as a medical specialty or discipline is still new to clinical practice and medical study. Many international medical schools have not yet adopted much of the recommended curricula put forth by the Consortium of Universities for Global Health in an attempt to standardize the core concepts and key principles for promoting best practices based on the available evidence [13, 14]. As global events affect everyone and impact human and health security, the requirement for all physicians in training, dentists and ancillary medical personnel to understand the basics of global health should be a key policy point. Increasing not only the awareness off, but also the core principles of global health, can be a multiplier of public health interventions, humanitarian response and overall evidence-based policy formation and programming. Human resources for global health will experience significant strain going forward, especially in resource poor areas, which are often found in fragile and failed states. This expertise must be cultivated through educational processes [15].

According to respondent freehand results, the international students surveyed showed a desire to learn more about pressing global health threats via a standardized format or curriculum. A third of respondents stated that the topics and ideas presented at the Prague Forum would influence or change their specialty and practice. Thus, further global health courses, programs, field activities and possibly a master’s program at Charles University should be developed and rooted in global health. Open-ended responses do speak for themselves of the diversity of students, student ideas about future global health programming and prioritization of global health threats.

## Limitations

The questionnaire did not collect data on ethnic background, country of origin, country of residence or the region or country that students planned to practice medicine or dentistry in upon graduation. By not capturing this data, the conclusions drawn about student responses related to the global health prioritization of key issues and their levels of interest in doing so may have been limited. The capacity building of state and regional institutions that promote health security can improve the response to public health crises. Future Global Health Forums may benefit from capturing more specific country or region statistics as it concerns clinical practice related to current global health issues.

## Conclusion

The universal promotion of global health and global health diplomacy in the medical undergraduate setting is still in its infancy. The global health paradigm is currently experiencing a moment of prolonged shock from epidemics, war and violence, climate change and resource shortages. This crisis situation may lead to decreased health security for millions of people. Medical services personnel will be asked to fulfill a greater role in responding to these challenges at clinical and policy levels. The core knowledge that healthcare personnel acquire through their training may dictate resource allocation and the prioritization of future responses to such crises. Medical practice relates not only to clinical acumen, and also gains more responsibility in terms of the policy regarding the response to global health threats; as such, medical curricula should be expanded to include more information on global health teaching. The Global Health Forum 2014 in Prague provided a venue for gauging current students’ thoughts and expectations as future physicians about the current most pressing global health topics. This brief commentary describes the results of a questionnaire completed by these students that addressed novel global health considerations.

## Abbreviations

HIV/AIDS: Human Immunodeficiency virus/Acquired Immunodeficiency Syndrome
ISIS: Islamic State of Iraq and the Levant/Islamic State of Iraq and Syria
NATO: North Atlantic Treaty Organization
NCD: Noncommunicable disease
PCGH: Prague Center for Global Health
PEPFAR: President’s Emergency Plan for AIDS Relief
TB: Tuberculosis
WHO: World Health Organization
